# Fasting blood glucose was linearly associated with colorectal cancer risk in the population without self-reported diabetes mellitus history

**DOI:** 10.1097/MD.0000000000026974

**Published:** 2021-08-27

**Authors:** Jingjing Wu, Huimin He, Qi Zhang, Yan Zhang

**Affiliations:** a Department of Oncology, the 900th Hospital of Joint Logistic Support Force, PLA, Fuzong Clinical College of Fujian Medical University, Fuzhou, Fujian, PR China; b Department of Natural Medicine, School of Pharmacy, Fujian Medical University, Fuzhou, Fujian, PR China.

**Keywords:** cancer risk, colorectal cancer, diabetes mellitus, fasting blood glucose

## Abstract

Fasting plasma glucose level was linearly associated with colorectal cancer (CRC) risk. However, the dose–response relationship between fasting blood glucose (FBG) and CRC risk was still uncertain.

A total of 11,632 patients without self-reported diabetes mellitus and colorectal polyps’ history were identified in the Korean Multicenter Cancer Cohort (1993–2005). The nonlinear relationship was estimated through a restricted cubic spline regression, and a two-piece-wise Cox proportional hazards model was further performed to calculate the threshold effect. Multiple imputation was used to control the bias from missing data.

Overall, 1.1% (n = 132) of participants were diagnosed with CRC in the follow-up duration. With a median follow-up duration of 12.0 years, participants with FBG ≥126 mg/dL were associated with higher CRC risk (adjusted hazard ratio [HR], 1.67; 95% confidence interval [CI]: 1.01, 2.76). Landmark analyses limited to long-term survivors demonstrated increased CRC risk with FBG ≥ 126 mg/dL in all subsets (≥3years: HR,1.93 (95% CI: 1.13–3.29); ≥5years: HR, 2.04 (95% CI: 1.–3.63); ≥10years: HR, 2.50 (95% CI: 1.19–5.25)). With FBG smoothly increasing before, the latter increased dramatically after the turning point (*P* for nonlinearity = 0.283). When FBG was increasing per mmol/L, HR was 1.07(95% CI: 0.90, 1.29) for FBG < 126 mg/dL and 1.27 (95% CI: 1.06, 1.53) for FBG ≥ 126 mg/dL. Besides, HR was 1.09 (95% CI: 1.02, 1.16) for the CRC risk.

In the population without self-reported diabetes mellitus and colorectal polyps’ history. FBG was linearly associated with CRC risk, especially for FBG over 126 mg/dL.

## Abstract 2

Supplemental Digital Content is available in the text

## 1 Introduction

Colorectal cancer (CRC) is the third most frequently diagnosed tumor worldwide, which is also the second leading cause of cancer-associated deaths, accounting for over 1.8 million new cases and 881,000 deaths in 2018.^[1]^ Therefore, identifying the CRC-related risk factors is an important way to increase CRC screening efficiency and reduce cancer-related deaths. The risk of developing CRC can be summarized as acquired factors and genetic factors. Furthermore, the previous studies have found the unhealthy dietary and lifestyles,^[2–4]^ such as a high-fat diet, obesity, and sedentary, are the key risk factors for CRC.

Diabetes mellitus (DM), which shares similar risk factors with CRC, has been regarded as an independent CRC-related factor.^[5,6]^ The underlying mechanisms involved in DM-related cancer are correlated with long-term hyperglycemia and hyperinsulinemia, and the former could activate IR and IGF-IR through activating the c-MYC-, PI3K-, MAPK-, or mTOR-pathway to impact cell survival and proliferation.^[7–9]^ Many observational studies have shown the positive relationship between DM and the risk of CRC. Moreover, individuals with DM have an approximately 20%–40% increased risk of developing CRC compared with nonDM.^[10–12]^ Besides, Park et al^[13]^ reports that fasting blood glucose level (FBG) over 126 mg/dL is associated with a 51% risk increase of CRC in the Korean population. Although a meta-analysis indirectly demonstrates a linear dose–response relationship between fasting plasma glucose and CRC risk,^[14]^ the association between FBG and CRC risk in the population without self-reported DM and colorectal polyps’ history is still uncertain.

To deal with this issue, we performed a secondary analysis to directly evaluate the dose–response relationship between FBG and CRC risk in the population without self-reported DM and colorectal polyps’ history.

## 2 Materials and methods

### 2.1 Data source

**Figure 1:**
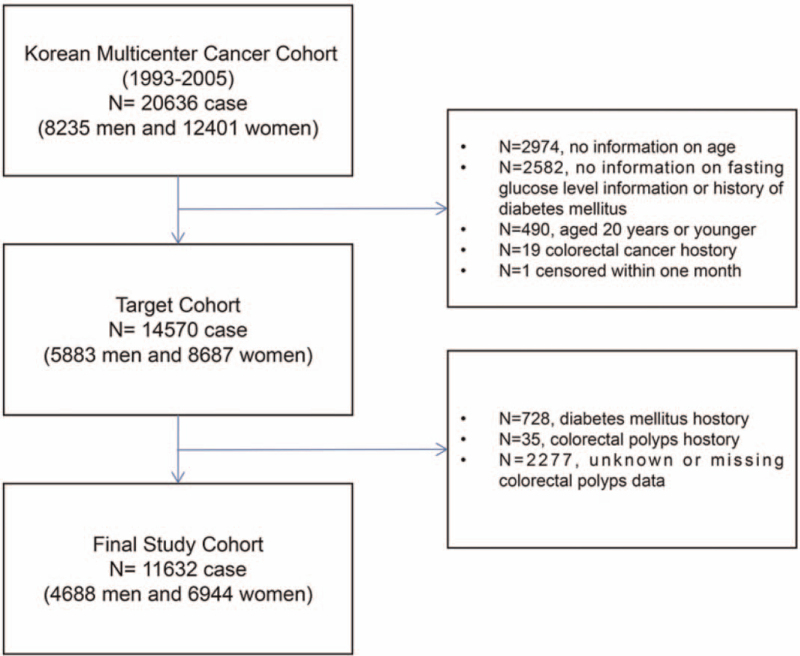
Inclusion/exclusion criteria. Data of the target cohort from the ‘Datadryad’ database are excluded by exclusion criteria I. Based on the target cohort, 11,632 cases are included in the final study cohort through exclusion criteria II.

This secondary analysis study was based on the raw data from Korean Multicenter Cancer Cohort, a large-scale prospective cohort study designed to investigate the relationship between exposures to environmental factors and lifestyle factors and cancer risk in Korea.^[15]^ Details of participates included were shown in Figure 1. This study (Institutional Review Board number: 1407-097-597) was approved by the Institutional Review Board at Seoul National University Hospital.^[13]^

Data were freely obtained from the ‘Datadryad’ database, a website that permits users to utilize original data freely. Authors of the initial study have waived all copyright and related ownership of these data. Therefore, we could use these data for secondary analysis without infracting on the authors’ rights.

### 2.2 Variables and endpoint definition

The details of data collecting (including age, education level, cigarette smoking, alcohol drinking habits, regular exercise status, and history of cancer) and measurement of fasting glucose concentrations were fully described in the previous study^[13]^ (see Doc S1, Supplemental Digital Content, http://links.lww.com/MD2/A336 which illustrates the variables and definition). Categorical variables included age (<55, ≥55 years), sex, body mass index (BMI) (<25, ≥25 kg/m^2^), formal education (no, yes), cigarette smoking (no, yes), alcohol drinking habits (no, yes), and regular exercise status (<7, ≥7 hours/week). The cutoff value (126 mg/dL) of FBG is based on clinical significance^[13]^ and the smooth plot of FBG and the logarithm of the hazard ratio (HR). Cancer-free survival was defined as the duration from recruitment to the end of follow-up. For the cases developing CRC, the end of follow-up was defined as the diagnosis date of CRC.

### 2.3 Statistical analyses

Firstly, Kaplan–Meier methods were used to estimate cancer-free survival rates for the FBG < 126 mg/dL and ≥126 mg/dL groups. Landmark analyses were performed for participants cancer-free, surviving a minimum of 3, 5, and 10 years from diagnosis to evaluate the immortal time bias. This method was also performed to calculate HRs in the different subgroups of longtime cancer-free survival. We also employed multivariable Cox regression analyses to test the association between FBG (vs <126 mg/dL) and CRC risk.

Next, to analyze the dose–response association between FBG and CRC risk, we applied a smooth curve to estimate the shape between FBG and CRC risk through a restricted cubic spline regression. FBG in the over 99th percentile group was excluded because of outlier data. Moreover, a smoothing plot was used to calculate the threshold effect of FBG on CRC risk based on the two-piece-wise Cox proportional hazards model. The inflection point was determined using the recursive method, where the maximum model likelihood was used. Furthermore, a log–likelihood ratio test was conducted to compare the one-line linear regression.^[16,17]^

Also, we built up the propensity score (PS) matching cohort to eliminate inherent differences between the FBG < 126 mg/dL and FBG ≥ 126 mg/dL group (see Figure S1 and Table S1, Supplemental Digital Content, http://links.lww.com/MD2/A337, which illustrates the baseline characteristics after propensity-matching FBG status and the distribution of propensity-matching FBG status groups after matching). Participants (FBG < 126 mg/dL vs ≥126 mg/dL) were matched in a 1:4 ratio based on PS (greedy-matching algorithm), with a caliper width equal to 0.05 of the standard deviation of the logit of the PS. To minimize bias, which may occur if confounders with missing data were excluded from the analysis. Finally, we used multiple imputation (MI), based on five replications and the Markov-chain Monte Carlo method in the R MI procedure, to account for missing data on the alcohol consumption history (no, yes), cigarette smoking history (no, yes). We then established an MI cohort to perform sensitivity analyses using a complete-case analysis.

All the analyses in our study were performed with R 3.4.3 (http://www.R-project.org) and EmpowerStats (http://www.empowerstats.com, X&Y Solutions, Inc., Boston, MA). *P* values less than .05 were considered statistically significant.

## 3 Results

### 3.1 Study population

In the previous study, 14,570 participants from 1993 to 2005 were included in the final analysis to investigate the association between FBG and risk of CRC. To better evaluate the impact of FBG on CRC risk in the average-risk population, we excluded the participants with self-reported DM history (n = 728), colorectal polyps (n = 35), and unknown colorectal polyps’ data (n = 2277). Moreover, 11,632 cases (4688 men and 6944 women), including 10,830 patients (93.11%) with FBG < 126 mg/dL and 802 (6.89%) cases ≥126 mg/dL, were included in the final analysis (Fig. 1). During a median of 11.3 years of follow-up, there were 132 colorectal cancer cases. The baseline characteristics of participants with FBG < 126 mg/dL or ≥126 mg/dL were presented in Table 1.

**Table 1:** General characteristics of participants according to fasting blood glucose levels, Korean Multicenter Cancer Cohort, 1993 to 2005.

	Fasting blood glucose levels (mg/dL)			
	<100	≥100, <126	≥126	*P* value
CRC case	89/8562	25/2268	18/802	.009
Age (yr)	53.1 ± 14.1	56.0 ± 13.4	58.0 ± 12.1	<.001
Sex				.052
Men	3414 (39.9%)	919 (40.5%)	355 (44.3%)	
Women	5148 (60.1%)	1349 (59.5%)	447 (55.7%)	
Body mass index (kg/m^2^)				<.001
<18.5	310 (3.6%)	80 (3.5%)	32 (4.0%)	
18.5–22	3282 (38.3%)	835 (36.8%)	245 (30.5%)	
23–24	1917 (22.4%)	532 (23.5%)	168 (20.9%)	
≥25	3053 (35.7%)	821 (36.2%)	357 (44.5%)	
Education level (yr)				<.001
Not formal education	1521 (17.8%)	536 (23.6%)	219 (27.3%)	
1–12	6719 (78.5%)	1659 (73.1%)	564 (70.3%)	
≥13	285 (3.3%)	66 (2.9%)	17 (2.1%)	
Missing	37 (0.4%)	7 (0.3%)	2 (0.2%)	
Moderate physical activity (h/wk)				.042
Never	2010 (23.5%)	600 (26.5%)	209 (26.1%)	
0.5–7	3574 (41.7%)	902 (39.8%)	319 (39.8%)	
7–21	1813 (21.2%)	487 (21.5%)	164 (20.4%)	
>21	999 (11.7%)	246 (10.8%)	101 (12.6%)	
Missing	107 (1.2%)	20 (0.9%)	3 (0.4%)	
Unknown	59 (0.7%)	13 (0.6%)	6 (0.7%)	
Alcohol consumption history				.029
Never	4689 (54.8%)	1294 (57.1%)	449 (56.0%)	
Former	449 (5.2%)	134 (5.9%)	50 (6.2%)	
Current	3330 (38.9%)	825 (36.4%)	300 (37.4%)	
Missing	94 (1.1%)	15 (0.7%)	3 (0.4%)	
Cigarette smoking history				.020
Never	5380 (62.8%)	1364 (60.1%)	464 (57.9%)	
Former	941 (11.0%)	274 (12.1%)	96 (12.0%)	
Current	2180 (25.5%)	620 (27.3%)	237 (29.6%)	
Missing	61 (0.7%)	10 (0.4%)	5 (0.6%)	

Data are Mean + SD/N (%). *P* values were based on the chi-square test for categorical measures and Kruskal–Wallis test for continuous measures. Fisher Exact test was performed if theoretical frequency <10. CRC = colorectal cancer.

### 3.2 Association of fasting blood glucose level on colorectal cancer risk

**Figure 2:**
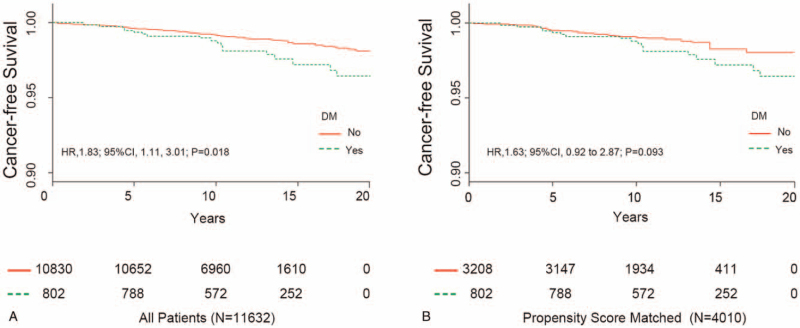
Cancer-free survival for patients with/without diabetes mellitus. (A) All patients. (B) Propensity score-matched patients. CI = confidence interval, DM = diabetes mellitus, HR, hazard ratio.

To evaluate the relationship between newly diagnosed DM on CRC risk, we firstly performed Kaplan–Meier methods to estimate cancer-free survival for the FBG <126 mg/dL and FBG ≥126 mg/dL groups. Compared with FBG ≥126 mg/dL groups, we found that participants with FBG <126 mg/dL had significantly longer cancer-free survival (*P* = .018, Fig. 2A) and unadjusted HR is 1.83 (95% CI: 1.11–3.01). According to the clinical and statistical significance, the variables in Table 2 were taken into multivariable Cox regression analyses, including age (<55, ≥55), sex (men, women), alcohol consumption history (no, yes), and cigarette smoking history (no, yes). After adjusting for these confounding factors, FBG over 126 mg/dL was associated with a 67% CRC risk increase (HR, 1.67; 95% CI: 1.01, 2.76; Table 3).

**Table 2:** Univariate analysis for risk factors of colorectal cancer in the Korean Multicenter Cancer Cohort, 1993 to 2005.

	Statistics	HR (95% CI)	*P* value
Age (yr)	54.00 ± 13.89	1.03 (1.02, 1.05)	<.0001
Age (yr)			
<55	5600 (48.14%)	1	
≥55	6032 (51.86%)	1.92 (1.34, 2.76)	.0004
Sex			
Men	4688 (40.30%)	1	
Women	6944 (59.70%)	0.55 (0.39, 0.77)	.0006
Formal education			
No	2276 (19.64%)	1	
Yes	9310 (80.36%)	0.74 (0.50, 1.09)	.126
Moderate physical activity (h/wk)			
<7	7614 (66.65%)	1	
≥7	3810 (33.35%)	1.02 (0.71, 1.47)	.918
Body mass index (kg/m^2^)			
<25	7401 (63.63%)	1	
≥25	4231 (36.37%)	1.09 (0.77, 1.55)	.625
Alcohol consumption history			
No	6432 (55.83%)	1	
Yes	5088 (44.17%)	1.65 (1.17, 2.32)	.004
Cigarette smoking history			
No	7208 (62.37%)	1	
Yes	4348 (37.63%)	1.50 (1.06, 2.12)	.021

Numbers that do not add up to 11,632 are attributable to missing data. HR, hazard ratio.

**Table 3:** Fasting blood glucose levels and multivariate HR with 95% CIs in colorectal cancer risk.

	CRC/participant	Nonadjusted	Adjusted model^∗^
FBG (vs <126 mg/dL)		1.82 (1.10, 2.99)	1.67 (1.01, 2.76)
FBG (mg/dL)			
<110	1	1	1
≥126	125/10625	1.82 (1.10, 3.00)	1.57 (0.95, 2.60)
After MI	132/11,632	1.80 (1.08, 3.01)	1.65 (0.99, 2.76)

∗ This model was adjusted for age (<55, ≥55), sex (men, women), alcohol consumption history (no, yes), and cigarette smoking history (no, yes). CI = confidence interval, CRC = colorectal cancer, FBG = fasting blood glucose levels, HR, hazard ratio, MI = multiple imputation.

**Figure 3:**
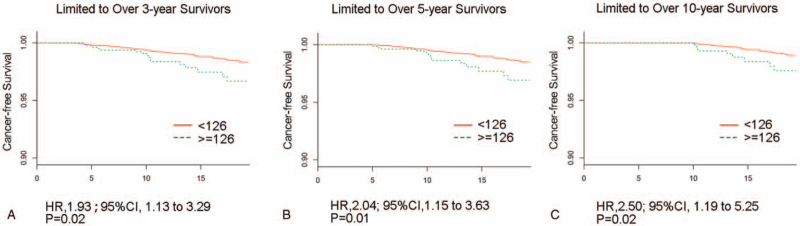
Landmark analyses of cancer-free survival for long-term (≥3, ≥5, ≥10 years) survivors. CI = confidence interval, HR, hazard ratio.

Next, landmark analyses evaluating the higher FBG impact for long-term cancer-free survivors of ≥3, ≥5, and ≥10 years were displayed in Figure 3. Overall, the FBG over 126 mg/dL group remained associated with lower OS for long-term survivors at each sequential landmark (all *P* < .05). Additionally, HR was 2.50 (95% CI: 1.19–5.25; Fig. 3C) in the ≥10 years subset of survival, which was higher than those ≥3 years (HR, 1.93; 95% CI: 1.13–3.29; Fig. 3A) and ≥5years (HR, 2.04; CI: 1.15–3.63; Fig. 3B).

**Figure 4:**
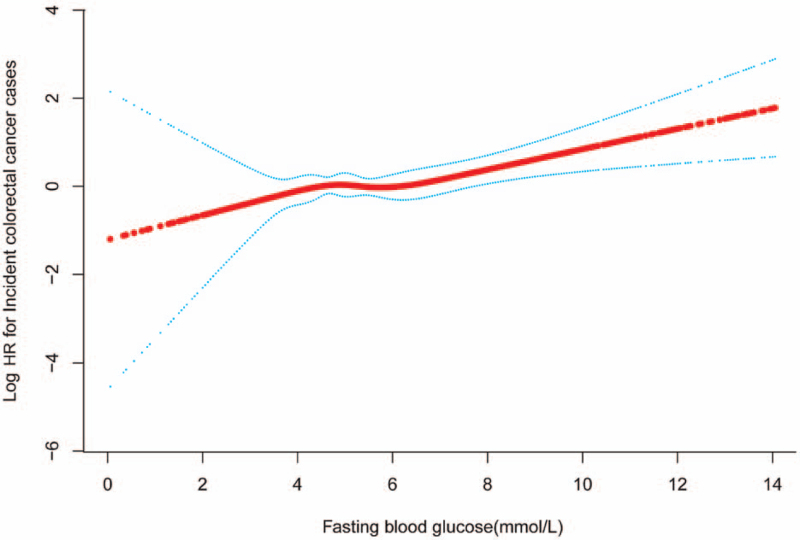
Smooth curves between fasting blood glucose levels and colorectal cancer risk.

In Figure 4, we found a nonlinear relationship between FBG and CRC risk. With FBG smoothly increasing before, the latter increased dramatically after the turning point (FBG = 126 mg/dL). When FBG was increasing per mmol/L, the HR was 1.07 (95% CI: 0.90, 1.29) for FBG < 126 mg/dL and 1.27 (95% CI: 1.06, 1.53) for FBG ≥ 126 mg/dL. However, the threshold effect of FBG on CRC risk was not significant (*P* for nonlinearity = .283). In the whole cohort, HR was 1.09 (95% CI: 1.02, 1.16) for the CRC risk, which was shown in Table 4.

**Table 4:** Threshold effect analysis for fasting blood glucose levels using two-piece-wise Cox proportional hazards model.

	CRC/population	Unadjusted HR (95% CI)
The one-line Cox proportional hazards model	132/11,587	1.17 (1.05, 1.29)
The two-piece-wise Cox proportional hazards model		
<126 mg/dL (increasing per mmol/L)	114/10,830	1.07 (0.90, 1.29)
≥126 mg/dL (increasing per mmol/L)	18/757	1.27 (1.06, 1.53)
*P* for log–likelihood ratio test		.283

A log–likelihood ratio test was used to compare the one-line linear regression. In this part, 45 participants were excluded for over the 99th percentile. CRC = colorectal cancer, HR, hazard ratio.

### 3.3 Sensitivity analysis

To eliminate differences between the groups, we built up a PS-matching cohort with 4010 cases (see Table 1). In the PS-matching cohort, however, CRC risk was not markedly higher in the FBG ≥ 126 mg/dL group vs the FBG < 126 mg/dL group (Fig. 2B; HR, 1.63; 95% CI: 0.92, 2.87; *P* = .093). The results were similar when participants with FBG ≥ 126 mg/dL compared with those who had FBG < 110 mg/dL (HR, 1.61; 95% CI: 0.96, 2.69), which was shown in Table 3. Finally, in Table 3, the results were still consistent after multiple imputation for the missing data.

## 4 Discussion

In this large-scale prospective study, we found that FBG was positively associated with CRC risk in the population without self-reported diabetes mellitus and colorectal polyps’ history, consistent with the previous studies’ CRC risk.^[5,6]^ Among the long-term survivors, the association between them became strengthening with the progressive duration of diabetes mellitus. Similarly, a trend of increased CRC risk was also observed in western patients with DM for the longer duration (4–8 years: HR, 1.19 [1.06–1.34]); >8 years: HR,1.28 [1.11–1.49]).^[11]^ Interestingly, a meta-analysis indirectly demonstrated the significant linear association between fasting plasma glucose and risk of colorectal cancer.^[14]^ Our study also found a significant linear dose–response relationship between fasting blood glucose and the incidence risk of colorectal cancer without self-reported diabetes mellitus and colorectal polyps’ history. In particular, we had a new finding that when FBG was over rather than less than 126 mg/dL, FBG increasing per mmol/L was 1.27 times higher risk of developing colorectal cancer.

Our current studies have several strengths. Firstly, this is a large-scale prospective cohort with a median follow-up of 12 years, which partially diminished the possibility of selection bias and reverse causation. Secondly, we utilized propensity score matching to balance the cohort on these potential confounders. Furthermore, multiple imputation was used to minimize bias for missing data. Thirdly, a smooth curve was applied to estimate the shape between FBG and hazard risk for CRC in the average-risk population.

Furthermore, several limitations deserved mention. Firstly, there were a few inevitable confounders in our cohort owing to the nature of observational research. Secondly, our conclusions were not pervasive because participants in the cohort were highly selected. In turn, our findings were beneficial to amplify the effectiveness of CRC screening in the average-risk population. Thirdly, we did not have access to complete information about the laboratory and clinical data, such as hemoglobin A1c and treatment (diet, oral medications, and insulin), potentially important confounders because of the secondary analysis. Fourthly, although the results were similar from sequential landmark analyses (≥3, ≥5, and ≥10 years), we still do not know the impact from the participants without self-reported colorectal polyps who were diagnosed as polyps-free 5 years or 10 years.

In summary, in this large prospective study, FBG was linearly associated with CRC risk in the population without self-reported diabetes mellitus and colorectal polyps’ history.

## Acknowledgments

We gratefully thank the Empower U team of the Department of Epidemiology and Biostatistics, X&Y solutions Inc. in Boston, to contribute to the statistical support.

## Author contributions

**Conceptualization:** Jingjing Wu, Huimin He, Yan Zhang.

**Data curation:** Jingjing Wu, Huimin He, Qi Zhang, Yan Zhang.

**Software:** Jingjing Wu, Huimin He, Yan Zhang.

**Supervision:** Yan Zhang.

**Validation:** Yan Zhang.

**Writing – original draft:** Jingjing Wu, Huimin He, Yan Zhang.

**Writing – review & editing:** Jingjing Wu, Huimin He, Qi Zhang, Yan Zhang.
